# Screening and transcriptomic analysis of anti-*Sporothrix globosa* targeting AbaA

**DOI:** 10.3389/fmicb.2025.1546020

**Published:** 2025-04-29

**Authors:** Ying Wang, Xiaoyan Wu, Xiyuan Fan, Chanxu Han, Fangliang Zheng, Zhenying Zhang

**Affiliations:** ^1^Academy of Life Science, Liaoning University, Shenyang, China; ^2^Department of Dermatology, University of Hong Kong Shenzhen Hospital, Shenzhen, China; ^3^Department of Dermatology, The Eighth Affiliated Hospital, Sun Yat-sen University, Shenzhen, China

**Keywords:** *Sporothrix globosa*, *abaA* gene, virtual screening, small molecule drugs, transcriptomics

## Abstract

**Introduction:**

Sporotrichosis is a fungal disease caused by a complex of *Sporothrix schenckii*, leading to chronic infections of the epidermis and subcutaneous tissue in both humans and animals.

**Methods:**

Through virtual screening targeting the key gene *aba*A to screen out the small-molecule drugs to treat Sporotrichosis. To further validate the antifungal activity of small-molecule drugs, growth curves, minimum bactericidal concentration (MBC), and minimum inhibitory concentration (MIC) for *Sporothrix globosa* (*S. globosa*) and *Sporothrix schenckii* (*S. schenckii*) were measured. In addition, we have done animal experiments to explore the function of the drugs. At the same time, qRT-PCR and transcriptome were used to verify the important role of *aba*A gene in *Sporothrix*.

**Results:**

Azelastine and Mefloquine effectively inhibit S. globosa and S. schenckii. MBC, and MIC for *S. globosa* and *S. schenckii* confirmed that both Azelastine and Mefloquine inhibited the growth of *S. globosa* and *S. schenckii*. Additionally, animal experiments demonstrated that Azelastine and Mefloquine reduced skin lesions in mice; post-treatment observations revealed improvements in inflammatory infiltration and granuloma formation. Through transcriptome analysis and qRT-PCR for validation, our findings demonstrate that the *aba*A gene plays a crucial role in regulating the attachment of the *Sporothrix* cell wall to the host matrix and in melanin regulation. Notably, when the *aba*A gene was inhibited, there was a marked increase in the expression of repair genes. These results emphasize the significance of the *aba*A gene in the biology of *Sporothrix*.

**Discussion:**

Two small-molecule drugs exhibit the ability to inhibit *Sporothrix* and treat sporotrichosis both in vitro and in murine models, suggesting their potential for development as therapeutic agents for sporotrichosis. And qRT-PCR and transcriptome results underscore the significance of the *aba*A gene in *Sporothrix*. Our results lay the foundation for the search for new treatments for other mycosis.

## 1 Introduction

Sporotrichosis is a fungal disease that leads to chronic fungal infections of the epidermis and subcutaneous tissue in both humans and animals. The pathogenic fungi involved are primarily a complex of *S. schenckii* (Chakrabarti et al., 2014; Hu et al., 2024). In the classical infection pathway, conidia initiate the fungal infection through the interaction between an implanted wound and decaying plant tissue, which results in the classification of sporotrichosis as a type of rot (Lopes-Bezerra et al., 2018; Lv et al., 2022; Liu et al., 2024). The main pathogens responsible for sporotrichosis include *Sporothrix schenckii, Sporothrix globosa*, and *Sporothrix brasiliensis* (Zu et al., 2020). In the northeastern region of our country, the predominant pathogenic strain is *Sporothrix globose*, however, this species remains underappreciated and poorly researched. To date, there is a limited amount of literature addressing its fundamental research and clinical implications (Nava-Pérez et al., 2022; Höft et al., 2022).

*Sporothrix globosa* is a dimorphic fungus to which immunocompromised individuals are particularly susceptible. This organism exists as mycelium in the environment at 25°C, from which conidia are released into the air, accompanied by fragments of the mycelium through wind and soil dispersion. When a wound comes into contact with soil or inhaled spores, the pathogen can enter the host at 37°C. Temperature changes can induce the transition to a new form-the yeast phase. Yeast forms are less readily recognized by the body’s immune cells compared to mycelial forms, rendering them less likely to be targeted by the immune response. Consequently, the dimorphic fungus *S. globosa* is not pathogenic in its mycelial phase but becomes pathogenic in its yeast phase. The variation in symptoms following infection is influenced by the individual’s immune status and differing genotypes. Overall, *S. globosa* exhibits weak virulence and typically presents with mild symptoms (Balkrishna et al., 2022).

Gene regulatory networks (GRN) control developmental events and play an important role in species evolution (Carroll, 2008; Levine, 2010; Smith et al., 2018), three DNA transcription factors, BrlA, AbaA, and WetA, regulate the developmental program of asexual fruiting bodies. BrlA activation program. AbaA regulates the development of conidiophore. In the *ΔabaA* mutant, the developmental program halts at the formation of the peduncle base. Consequently, the mutant conidiophores deform into globular structures that are dispersed across the rod-shaped peduncle base, resembling the appearance of an abacus (Clutterbuck, 1969). WetA controls the maturation of asexual spores (Etxebeste et al., 2019; Yu, 2010). Regarding research on the DNA-binding domain of the AbaA transcription factor, Borneman et al. cloned a homolog of the *Aspergillus nidulans abaA* gene, which encodes the ATTS/TEA DNA-binding domain transcriptional regulator, and transformed it into *Penicillium marneffei* (Borneman et al. 2000). Their findings indicate that the *abaA* gene plays a crucial role in the developmental process of transitioning from the mycelial phase to the yeast phase. Additionally, Alex et al. also demonstrated that the amino acid sequence of the AbaA transcription factor contains an ATTS/TEA DNA binding motif (Andrianopoulos and Timberlake, 1994). Targeted deletion of AbaA blocks asexual development at 25°C prior to spore production, resulting in abnormal conidia with repeat terminal cells. Furthermore, the *abaA* deletion strain fails to properly switch from multinucleated filamentous forms to mononucleated yeast cells at 37°C. Many studies have found the DNA-binding domain of *abaA* gene is conserved and plays an important role in dimorphic switching (Andrianopoulos and Timberlake, 1994).

In recent years, the emergence of multi-resistant pathogens has made fungal infections increasingly difficult to treat. Consequently, strategies for managing hospital infections and opportunistic infections have garnered significant attention within the scientific community (Perlroth et al., 2007; Badiee and Hashemizadeh, 2014). Currently, treatment options for sporotrichosis are limited, and emerging resistance is a concern (Zhang et al., 2024; Brown-Elliott et al., 2001), there was no improvement observed after 16 months of treatment with terbinafine, fluconazole, and itraconazole. Consequently, there is an urgent need for new treatments for sporotrichosis.

Molecular docking is an important technology in computer-aided drug design, which is widely used in new drug development (Farha and Brown, 2019). The process of researching and developing new drugs is lengthy and requires substantial financial resources. Currently, the issue of fungal drug resistance is becoming increasingly severe. In recent years, a notable trend has emerged in the development of new drugs that involves the integration of various disciplines, particularly the combination of biology, computer science, and chemistry. With advancements in computer science, computer-aided drug design has become a prominent focus in the development of new drugs, especially through the use of molecular docking techniques (Swamidass, 2011; Jadamba and Shin, 2016). The development of new drugs is a lengthy and inefficient process, primarily due to challenges related to identifying new targets, ensuring safety, and managing significant associated costs. Consequently, repurposing drugs that have already been approved for other human conditions may offer a more expedient approach to discovering new antifungal agents. In various areas of clinical research, drug repurposing has emerged as a strategy to accelerate the development of new therapies, often utilizing drug-based phenotypic screening methods or high-throughput screening of FDA-approved drug libraries (Park, 2019). In this context, target recognition can be used to target new diseases for drug repurpose (Ma et al., 2022). So this study will look for new ways to treat sporotrichosis through a combination of bioinformatics and traditional experiments. The *abaA* gene plays a crucial role in the dimorphic switch of *Sporothrix*, and the downstream virulence factors, along with other related genes regulated by the *abaA* gene, were also investigated using bioinformatics and transcriptomic methods.

## 2 Materials and methods

### 2.1 Screening of small molecule drugs targeting AbaA protein

The target was AbaA protein, a key dimorphic switch protein of *S. schenckii*, used the Robetta^1^ to predict the three-dimensional structure of AbaA, the DNA binding domain of AbaA protein was found by bioinformatics analysis. Then, the DNA-binding domain portion of the model’s highest-quality three-dimensional structure was truncated for binding pocket prediction. The grid box was determined by aligning the structure of the AbaA DNA domain, and the x, y, and z-coordinates of the grid box (*x* = 67.154, *y* = 85.033, *z* = −92.830; *x* = 58.693, *y* = 85.350, *z* = −82.023) were determined. AutoDock Vina was then used to perform bulk molecular docking in the FDA-approved small molecule database, the docking results were comprehensively analyzed in terms of binding energy, price, pharmacodynamics and side effects (Moreira et al., 2021; Haozhen et al., 2023).

### 2.2 Fungal strain and culture conditions

The strain of *S. globosa*, *S. schenckii* used was maintained at the Research Center for Pathogenic Fungi, Liaoning University, China. To obtain a mycelial culture, the *S. globosa*, *S. schenckii* was inoculated onto Sabouraud dextrose agar (SDA) solid medium (10 g/L tryptone, 40 g/L glucose, 15 g/L agar) and incubated at 25°C. To induce the switch of *S. globosa* and *S. schenckii* from the mycelial phase to the yeast phase, mycelial culture was enriched and transferred to brain-heart infusion (BHI) liquid medium, which was incubated at 37°C.

### 2.3 *In vitro* antifungal susceptibility

Antifungal susceptibility testing was performed using the proto-cols described in the CLSI document. *Sporothrix* was cultured at 25°C for 4 days, followed by filtration and centrifugation. The spores were resuspended in autoclaved BHI liquid medium, adjusting the spore concentration to 1 × 10^5^ CFU/mL. In a 96-well plate, 200 μL of the test drug working solution was added to Well 1, while 100 μL of BHI liquid medium was added to Wells 2–11. Well 12 received 200 μL of BHI liquid medium as a negative control. A 100 μL aliquot from Well 1 was transferred to Well 2 and gently mixed by pipetting up and down. This serial dilution process was repeated for Wells 3–10. After mixing Well 10, 100 μL of the supernatant was discarded. Finally, 100 μL of the prepared spore suspension (from Step 2) was added to each well. MIC and MBC results were read by visual inspection and from the readings of the cell optical density at an absorbance of 625 nm (OD_625_) (Joao et al., 2020; Van Cutsem et al., 1994).

### 2.4 Measurement of growth curve

The *Sporothrix* cultured at 25°C with shaking at 150 rpm for 4 days were transferred to 50-mL sterile centrifuge tubes and centrifuged at 8,000 rpm for 5 min. The supernatant was then discarded. The mycelium was diluted in BHI medium, in groups, the final concentration of the drug was 50 μg/mL by adding quantitative *Sporothrix* suspension and DMSO-dissolved small molecule drug in groups. 37°C for 96 h, OD_625_ was measured and photographed under bright-field microscopy at 40 × magnification every 12 h, the experiment was repeated three times for each group.

### 2.5 Murine model of sporotrichosis

Male 8-week-old KM mice were purchased from Liaoning Changsheng Biotechnology Co. Ltd., permit No. SCXK (Liao)2020–0001. The mice in all groups were injected intraperitoneally with cortisol solution every other day for 1 week before inoculation with sporotrichosis suspension. 20 mg/kg, after 1 week it was changed to every 2 days, gavage administration was started after successful modeling (Feng et al., 2010; Boyce et al., 2011). All groups of mice had their abdominal skin shaved with a razor before being injected with *Sporothrix* suspension, 0.1 mL *Sporothrix* suspension was injected intradermically into one of the hair-removal sites with a 1 mL syringe, it contains about 1 × 10^7^ spores.

### 2.6 Small molecule drugs treatment

After modeling, Daily Gavage was started. Mice were randomly assigned to two treatment cohorts: the Azelastine treatment group and Mefloquine treatment group. These cohorts were further stratified into high-dose (Azelastine: 6 mg/kg/day, Mefloquine: 20 mg/kg/day), low-dose (Azelastine: 3 mg/kg/day, Mefloquine: 3.8 mg/kg/day), and a control group receiving 0.5% carboxymethylcellulose sodium salt.

### 2.7 Histological examination of the skin

Following drug administration, the mice were euthanized through cervical dislocation and immersed for 5 min in a 5% phenol solution for disinfection purposes. The specimens were then rinsed three times with autoclaved sterile distilled water and placed on a sterile dissecting board under aseptic conditions. Skin lesions were excised and immediately fixed in a 4% paraformaldehyde solution for histopathological processing. Skin lesion samples were sent to Jijia for HE staining, the infiltration of inflammatory cells and the formation of granuloma were observed and analyzed.

### 2.8 Statistical analysis

The study groups were compared statistically using the SPSS 23.0. Significance for all statistical tests is shown in the figures for *p* < 0.05, *p* < 0.01, and *p* < 0.001.

### 2.9 Ethics statement

The experiment was conducted in strict accordance with the Guide for the Care and Use of Laboratory Animals.

### 2.10 cDNA library construction and sequencing

Total RNA was extracted from the mycelial phase of a 48-h culture, the yeast phase of 48-h induction and the liquid of 48-h culture following the addition of Azelastine. The total amount and purity of the extracted RNA were assessed. After qualification, eukaryotic mRNA was enriched using magnetic beads with Oligo (DT). Fragmentation buffer was subsequently added to cleave the mRNA into shorter fragments. Using the mRNA as a template, single-stranded cDNA was synthesized with random primers, followed by the addition of RNase H to produce double-stranded cDNA. The resulting double-stranded cDNA was purified, its ends were repaired, a tail was added, and sequencing linkers were connected. Fragments were selected using AMPure XP beads. Finally, PCR amplification and purification were performed to obtain the final library. The libraries were quality-checked, and those meeting the criteria were subjected to PE150 sequencing using the Illumina HiSeq 2,500 high-throughput sequencing platform.

### 2.11 Transcriptome analysis

First, the quality of sequencing data was evaluated using FASTP (Chen et al., 2018) software. Following quality control, the sequencing data were aligned with ribosome sequences from the NCBI RefSeq (Pruitt et al., 2007) and RFAM (Kalvari et al., 2021) databases utilizing Bowtie 2 (Langmead et al., 2019) software. The alignment results were statistically processed using samtools (Danecek et al., 2021) and subsequently compared to the reference genome with Bowtie 2 (Langmead et al., 2019). Transcript assembly was performed with StringTie (Pertea et al., 2015) software, leveraging available reference information, followed by quantitative analysis of gene expression levels. Once the transcript read counts were obtained, they were converted to gene read counts using the R package maximport (Soneson et al., 2015). After acquiring read counts for all samples, we employed the differential analysis software DESEQ2 (Love et al., 2014) to conduct differential expression analysis of genes. For this analysis, we utilized the GO, KEGG, and EggNOG databases for functional annotation, functional enrichment, and GSEA analyses.

### 2.12 Real-time quantitative PCR

Approximately 100 mg of *S. globosa* mycelial-phase cells and yeast-phase cells were collected and rapidly frozen in liquid nitrogen. The samples were subsequently ground under continuous liquid nitrogen cooling using a mortar and pestle. Total RNA was extracted from both phases using the TRIzol Reagent Kit (Vazyme Biotech Co., Ltd., China) in accordance with the manufacturer’s protocol. Quantitative real-time PCR was conducted on the StepOnePlus system. Following normalization with the 18S rDNA reference gene, the relative expression levels of the target gene between the yeast-phase and mycelial-phase were compared and analyzed using the 2^(-ΔΔCt) method.

## 3 Results

### 3.1 Discovery of anti—*S. globosa* entry inhibitors among candidates in the FDA approved drug library

**Figure 1:**
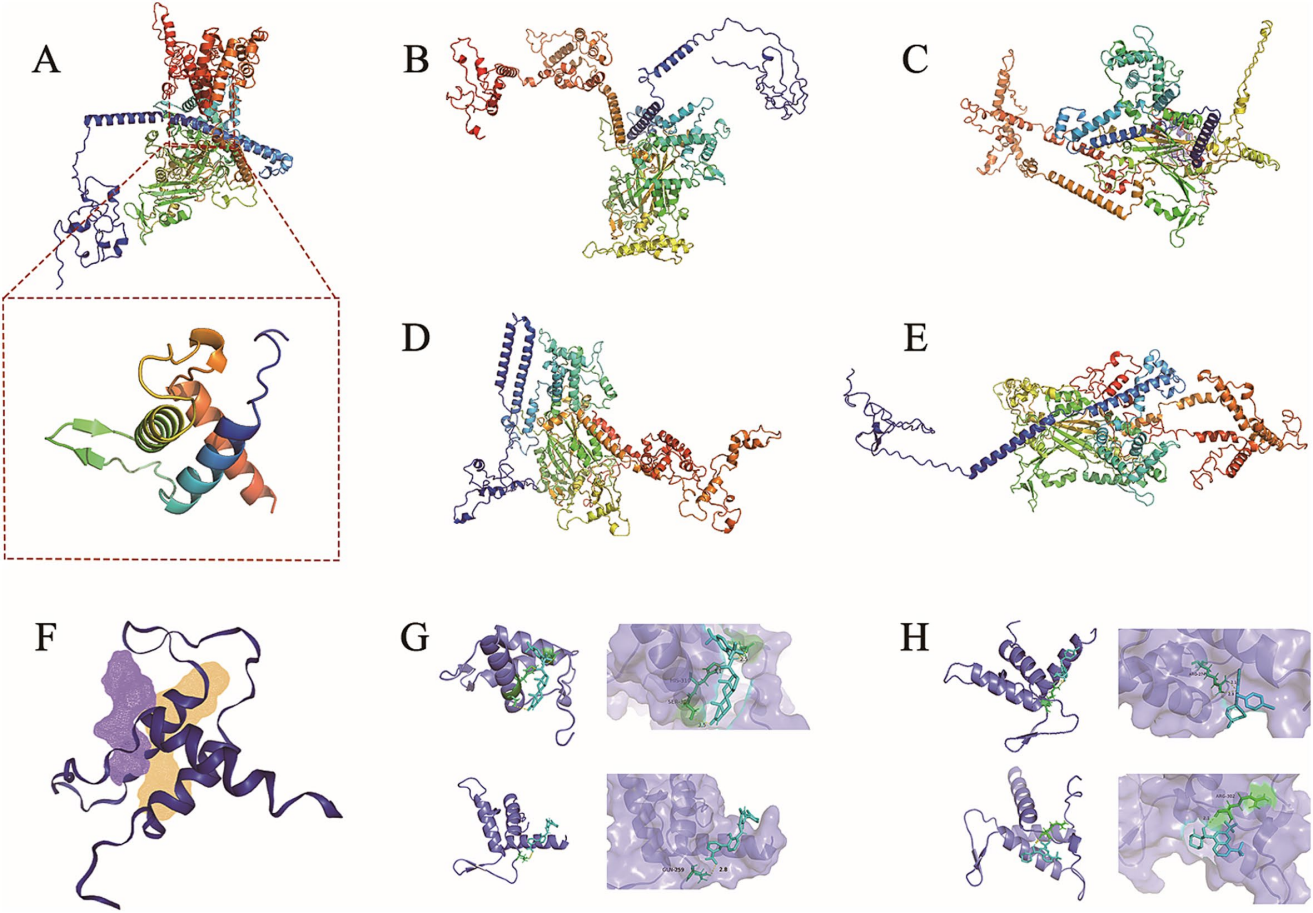
AbaA three-dimensional structure prediction and docking results display. **(A–E)** The three-dimensional structure of AbaA protein was predicted using Robetta, the three-dimensional structure of AbaA DNA-binding domain is shown under **(A)**. **(F)** Use DoGSiteScorer to predict the protein-binding pocket. **(G,H)** The four graphs are Avodart, Eltrombopag, Azelastine, Mefloquine and AbaA DNA binding domain docking results.

The *abaA* gene sequence of *S. globosa* utilized in this study was obtained through sequencing conducted in our laboratory. The three-dimensional structure of this gene is currently unknown, prompting a search for similar proteins to facilitate further investigation. In order to search for similar proteins of AbaA, we first searched for similar genes of *abaA* (Table 1), HMPREF1624, which exhibited the highest total score and sequence similarity, was selected and subsequently searched in the UniProt database (Supplementary Table S1). This protein contains a DNA-binding domain known as TEA (Supplementary Table S2), which is identical to that of the AbaA protein. We used the RoseTTAFold module in the Robetta server to predict the three-dimensional structure of the AbaA protein (Figures 1A–E), the five predicted models are evaluated in SAVES v6.0 (Table 2), we finally chose the highest score Result-1 for follow-up processing (Moreira et al., 2021; Cho et al., 2009; Ma et al., 2022). We utilized PyMOL to truncate the AbaA protein’s DNA-binding domain from the model Result-1 (Figure 1A). The binding pockets of this domain were predicted using DoGSiteScorer (Table 3, Figure 1F). We then employed AutoDock Tools to preprocess the top two pockets, setting the size and coordinates of the docking box. Subsequently, we conducted batch molecular docking using AutoDock Vina (Supplementary Table S3). We compared the binding energies and analyzed the primary efficacy and side effects of the selected small molecule drugs. Ultimately, we chose four small molecule drugs, Avodart, Eltrombopag, Azelastine, and Mefloquine, for further study (Figures 1G,H).

**Table 1:** BLAST comparison results.

Description	Max score Total score	Query cover	E value	Percent identity	Accession
HMPREF1624_03084	1,621	99%	0.0	94.11%	ERS99720.1
SPSK_08393	1,608	99%	0.0	93.97%	XP_016588585.1
transcription factor	1,606	99%	0.0	94.38%	XP_040619892.1

**Table 2:** Results of model quality evaluation.

Result	Verify 3D	Procheck	Whatcheck	Errat	Prove
1	Fail	Pass:3	Green:29	91.6514	Fail
2	Fail	Pass:3	Green:29	91.0009	Warning
3	Fail	Pass:2	Green:27	89.7179	Warning
4	Fail	Pass:2	Green:28	91.7498	Fail
5	Fail	Pass:2	Green:25	87.3733	Fail

**Table 3:** Combine pocket predictions.

Pocket number	Volume (Å^3^)	Surface (Å^2^)	Drug Score	Simple Score
1	499.78	912.12	0.72	0.36
2	436.16	921.93	0.76	0.34
3	227.65	316.54	0.55	0.13

### 3.2 Azelastine and Mefloquine have *in vitro* antifungal activity against the *S. globosa* and *S. schenckii*

**Figure 2:**
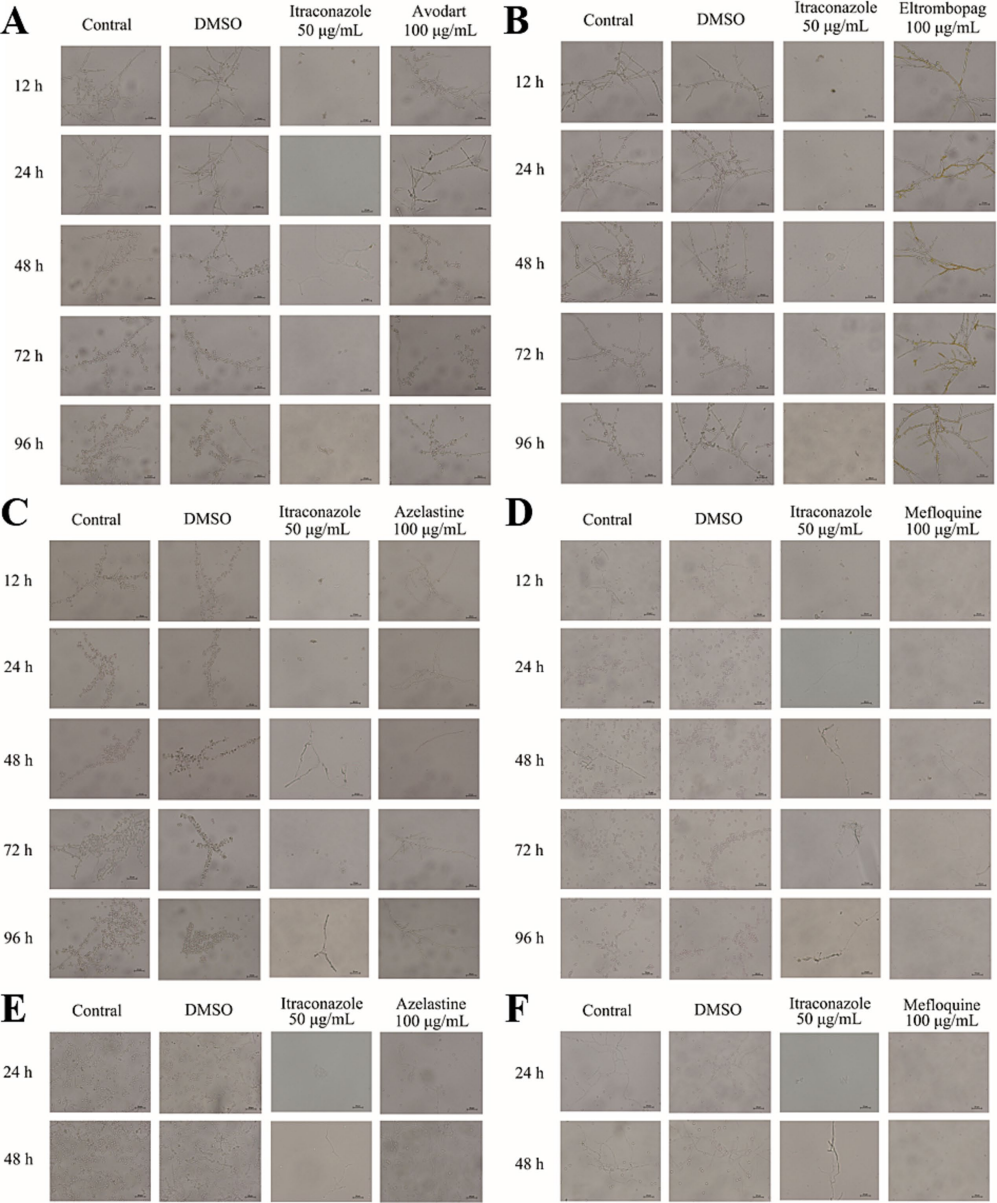
Antifungal activity of the candidate against *Sporothrix globose* and *Sporothrix schenckii* under bright-field microscopy at 40 × magnification. **(A)** *In vitro* antifungal activity of Avodart against *Sporothrix globose*. **(B)** In vitro antifungal activity of Eltrombopag against *Sporothrix globose*. **(C)** In vitro antifungal activity of Azelastine against *Sporothrix globose*. **(D)** In vitro antifungal activity of Mefloquine against *Sporothrix globose*. **(E)** In vitro antifungal activity of Azelastine against *Sporothrix schenckii*. **(F)** In vitro antifungal activity of Mefloquine against *Sporothrix schenckii*. The test concentration was 50 μg/mL, 100 μg/mL, and the selected concentration was 100 μg/mL. The amount of DMSO is the amount of candidate drugs.

In our previous studies, *abaA* gene expression was upregulated during the dimorphic switch of the *S. schenckii* from the mycelial phase to the yeast phase, and the deletion of this gene causes *S. schenckii* to become less resistant to all kinds of stress, these results suggest that the gene is critical for *S. schenckii* dimorphic switch (Zheng et al., 2021). Therefore, it is reasonable to speculate that this gene is also important for the dimorphic switch of *S. globosa*. Consequently, we suspect that AbaA is also upregulated during the yeast phase of *S. globosa*. To explore the antifungal activity of Azelastine and Mefloquine, the selected drugs were administered to *S. globosa* and the *S. schenckii* phase reversal process respectively, in order to observe their effects on the dimorphic switch of *S. globosa* and *S. schenckii*. The results indicate that Avodart and Eltrombopag have no effect on either fungus (Figures 2A,B). In contrast, Azelastine and Mefloquine showed inhibitory effects on both *S. globosa* and *S. schenckii* when compared to the control group and DMSO solvent control group (Figures 2C–F). When Azelastine and Mefloquine were added, the conidia of *S. globosa* dropped off and did not grow. Both Azelastine and Mefloquine significantly inhibited the growth and spore production of *S. globosa* and *S. schenckii*. In the control and solvent control groups, dimorphic switch of *S. globosa* and *S. schenckii* occurred, there was no phase inversion in the Azelastine and Mefloquine groups. Thus, both small-molecule drugs inhibited the growth and dimorphic switch of *S. globosa* and *S. schenckii*.

### 3.3 Determination of fungal growth curves, MIC and MBC of Azelastine and Mefloquine

**Figure 3:**
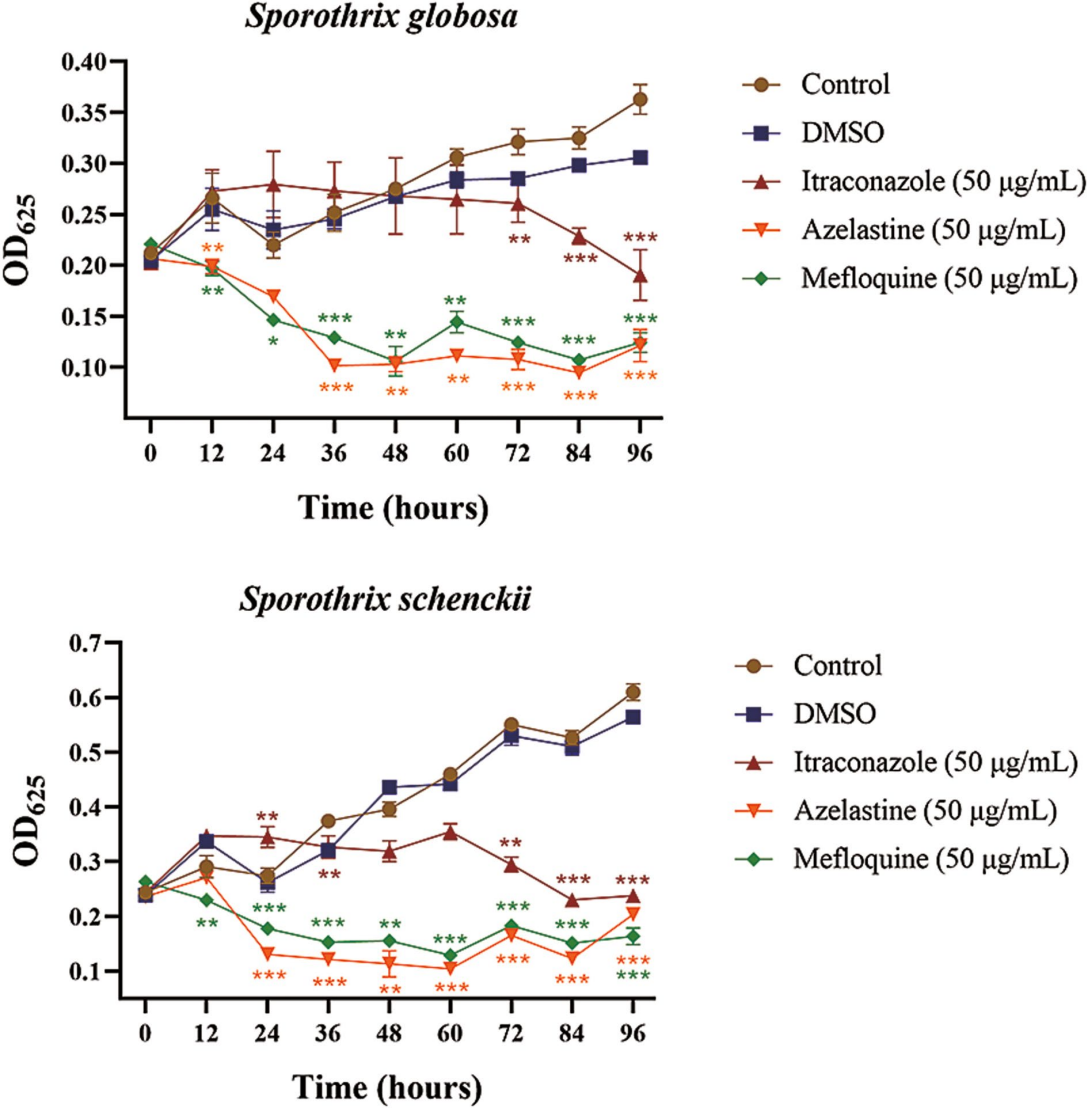
The growth curves of *Sporothrix globose* and *Sporothrix schenckii* were inhibited by small molecule drugs. **(A)** The growth curves of the small molecule drugs for the inhibition of *Sporothrix globosa*. **(B)** The growth curves of the small molecule drugs for the inhibition of and *Sporothrix schenckii*. DMSO group was solvent control group and Itraconazole group was positive control group. *, *p* < 0.05; **, *p* < 0.01; ***, *p* < 0.001. Data processing used SPSS 23.0 for Ducan’s multiple comparison test to analyze its significance.

Given the subjective nature of morphological observations, we aimed to further investigate the inhibitory effects of Azelastine and Mefloquine on *S. globosa* and *S. schenckii*. To achieve this, we determined the growth curves for these small molecule drugs in relation to the inhibition of both *S. globosa* and *S. schenckii*, and we determined the MIC and MBC of the two small molecule drugs. The addition of Azelastine and Mefloquine significantly inhibited the growth of *S. globosa* and *S. schenckii* compared with the control group and DMSO solvent control group (Figure 3). Although the measured MIC and MBC values do not show a small-dose advantage over other fungicide (Table 4), these agents are considered safer than antibiotics and are administered at lower doses compared to Itraconazole alone. Both drug stents can also be optimised for the development of new small-molecule drugs to treat sporotrichosis if the clinical dose is higher than the safe range.

**Table 4:** Azelastine and Mefloquine inhibited MIC and MBC of *Sporothrix globose, Sporothrix schenckii*.

Drug	MIC (μg/mL)	MBC (μg/mL)
Azelastine-*Sporothrix globose*	25	50
Mefloquine-*Sporothrix globose*	12.5	25
Azelastine-*Sporothrix schenckii*	6.25	50
Mefloquine-*Sporothrix schenckii*	6.25	25

### 3.4 Azelastine and Mefloquine are effective for sporotrichosis

**Figure 4:**
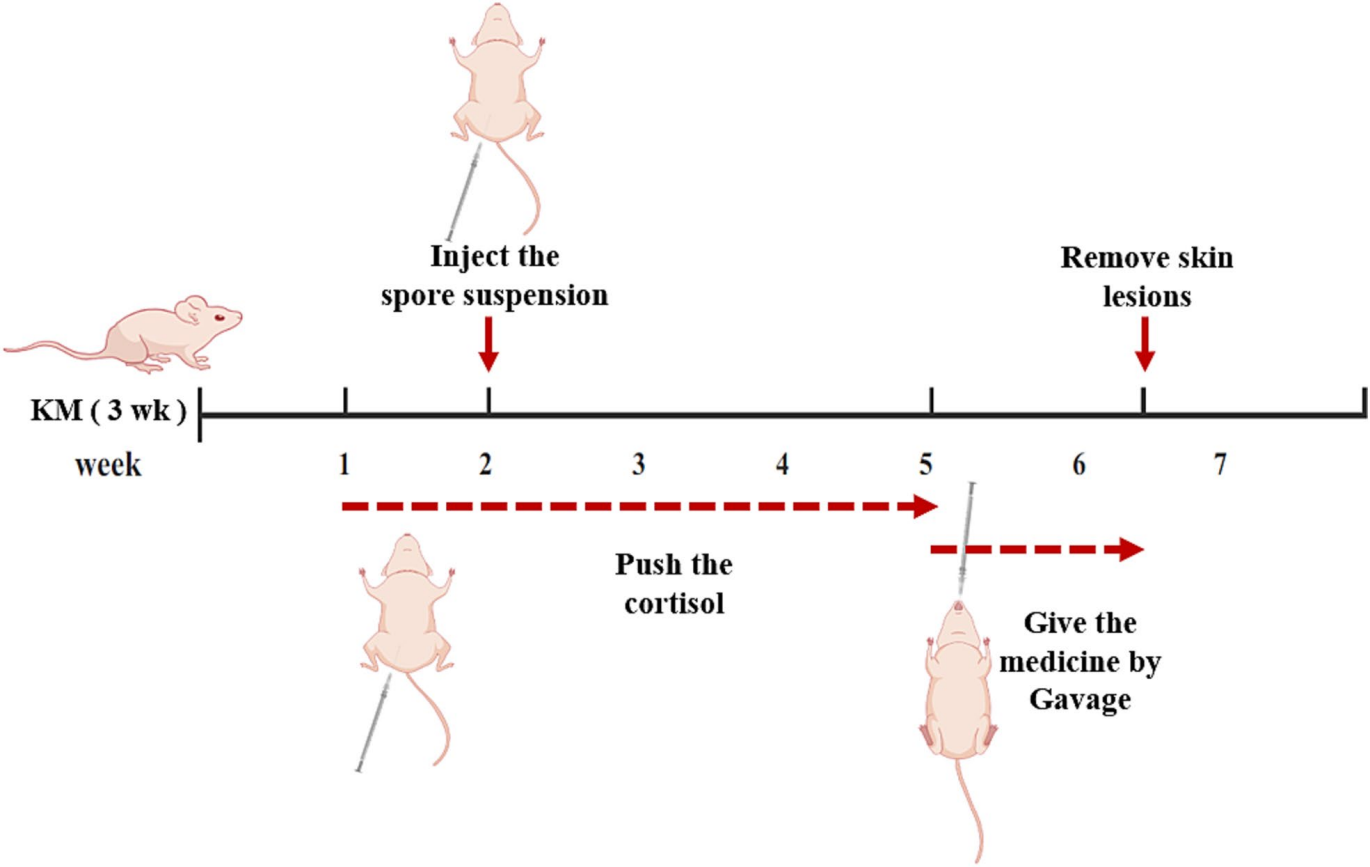
Mouse model and treatment process. After a week of acclimation, the mice were injected with a cortisol solution for immunosuppression, after 1 week of inhibition, the mold was made by injection *Sporothrix* suspension, meanwhile, immunosuppression was continued. Three weeks after successful modeling, the rats were treated with medicine. After 10 days of administration, mice were killed and skin lesions were examined.

**Figure 5:**
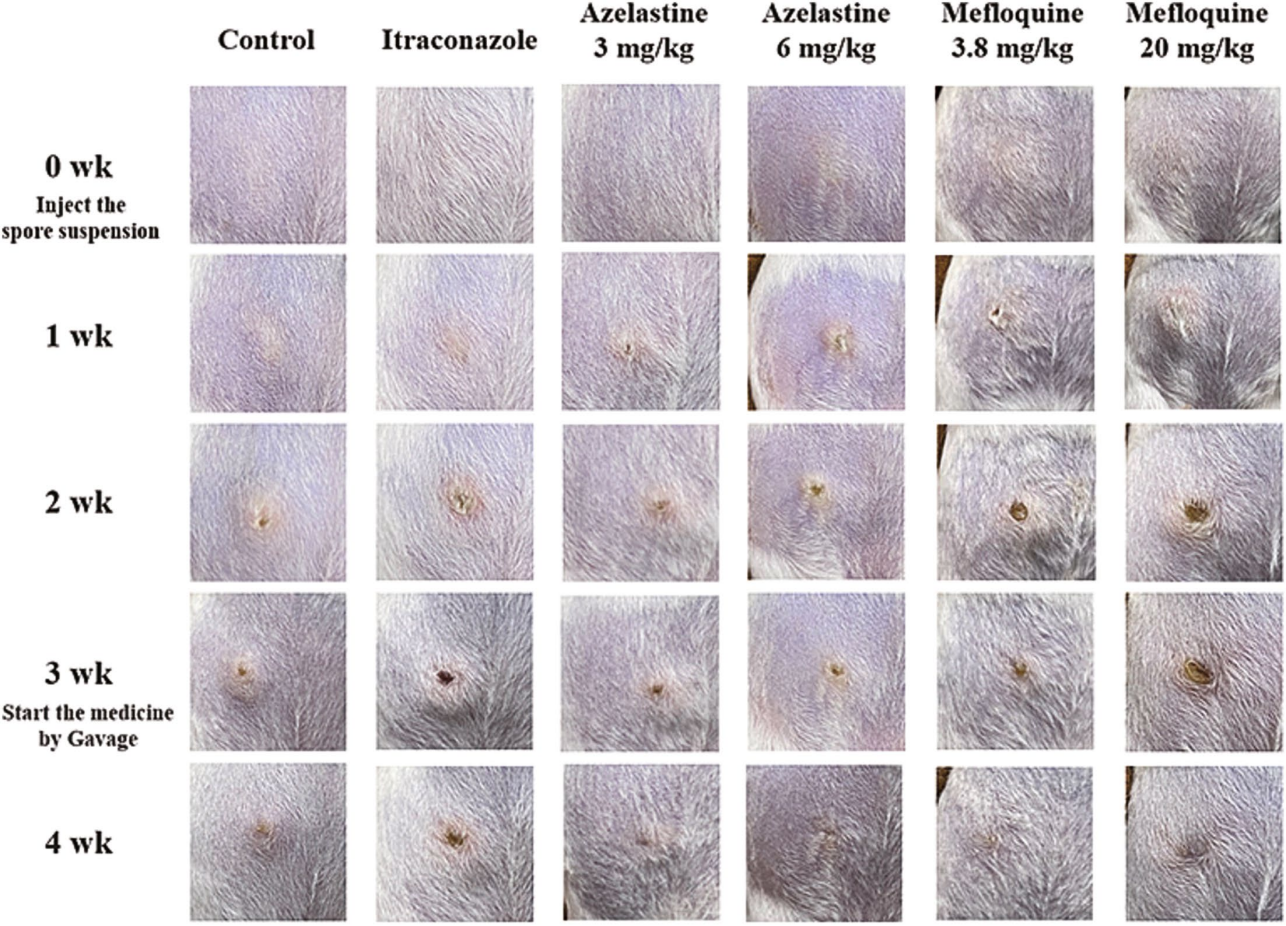
Pictures of mouse model and treatment process. Intradermal injection of *Sporothrix* suspension into the abdomen of mice produced varying degrees of skin lesions, some grow nodules, some ulcers, or cysts.

**Figure 6:**
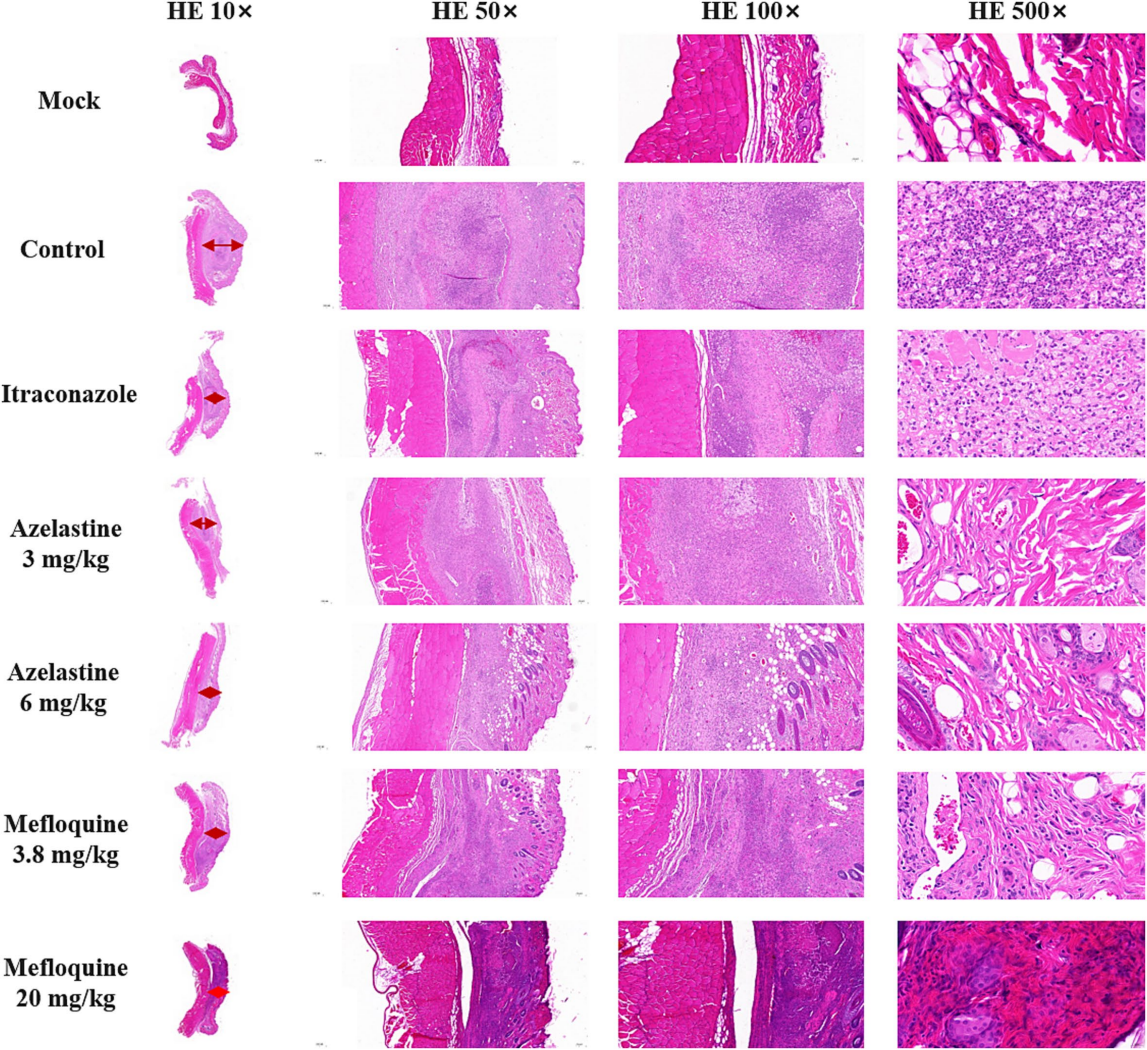
HE staining results of skin lesions in mice. The Mock group was the result of HE staining of healthy skin, the red arrow was granuloma, control group was not given medicine after successful modeling. The width of granuloma in control group was 3,320 μm; the width of granuloma in Itraconazole group was 1,460 μm; the width of granuloma in Azelastine 3 mg/kg group was 1,880 μm; the width of granuloma in Azelastine 6 mg/kg group was 1,480 μm; the width of granuloma in Mefloquine 3.8 mg/kg group was 1,940 μm; the width of granuloma in Mefloquine 20 mg/kg group was 1,251 μm. (Granuloma width was measured by software CaseViewer).

We verify the efficacy of Azelastine and Mefloquine in the treatment of sporotrichosis at the animal level. The model was established by intradermal injection of *Sporothrix* spore suspension in mice (Figure 4) Intradermal injection of spore into the abdomen of mice produced varying degrees of skin lesions, some grow nodules, some ulcers, or cysts. For some nodules, the pus was collected by gastric perfusion. The pus was dipped into a high-temperature sterilized cotton swab and diluted with sterile distilled water. 40 μL of pus was spread evenly on SDA medium and cultured at 25°C for 4 days, microscopy confirmed *S. schenckii* (Supplementary Figure S1). Inflammatory ulcers and nodules began to appear in the skin of mice 1 week after intradermal injection, the morphology of the lesions was analyzed by reviewing the literature, and the pus was purified and cultured, microscopic observation showed that the lesions were Sporotrichosis. The Sporotrichosis was most severe in the third week, when the drug was given intragastrically for 10 days, the nodules in the Itraconazole positive control group and the Azelastine and Mefloquine groups became smaller and the crusts were improved (Figure 5). In comparison to the mock group, the control group exhibited larger granulomas and a greater infiltration of inflammatory cells. Notably, the granulomas in the positive control group were smaller than those in the control group. The granulomas observed in both the low and high dose Azelastine groups were wider than those in the positive control group, yet smaller than those in the control group; they were dispersed and did not form a cohesive mass, with milder inflammatory cell infiltration compared to the control group. In contrast, the inflammatory infiltration in both the low and high dose Mefloquine groups did not show improvement relative to the control group; however, the granulomas were significantly smaller, with the inflammatory cells remaining dispersed and not coalescing (Figure 6). The granuloma width of the control group was significantly different from that in both the positive control group and the treatment group (Table 5). Statistical analysis of inflammatory cell statistics showed that there were significant differences between the control group and the positive control group (Table 6).

**Table 5:** Results of granuloma width statistical analysis.

Group	Granuloma width (μm)
Control	2588.9 ± 423.4^b^
Itraconazole	1446.7 ± 418.5^a^
Azelastine 3 mg/kg	1059.3 ± 699.8^a^
Azelastine 6 mg/kg	1136.9 ± 224.4^a^
Mefloquine 3.8 mg/kg	1054.7 ± 138.6^a^
Mefloquine 20 mg/kg	911.4 ± 253.2^a^

Different lowercase letters indicate significant difference (*P* < 0.05). SPSS 23.0 was used to analyze the significance of Ducan multiple comparison test.

**Table 6:** Results of inflammatory cell count were analyzed statistically.

Group	Inflammatory cell count
Control	566.9 ± 72.3^b^
Itraconazole	255.2 ± 29.7^a^
Azelastine 3 mg/kg	263.1 ± 93.4^a^
Azelastine 6 mg/kg	273.6 ± 85.5^a^
Mefloquine 3.8 mg/kg	333.1 ± 71.7^a^
Mefloquine 20 mg/kg	222.8 ± 11.9^a^

Different lowercase letters indicate significant difference (*p* < 0.05). SPSS 23.0 was used to analyze the significance of Ducan multiple comparison test. Inflammatory cells were counted using software Image-J.

### 3.5 The regulatory effect of the *abaA* gene on downstream virulence factors was analyzed using bioinformatics

To further investigate the regulation of the *abaA* gene on these virulence factors, we reviewed the literature to identify the downstream virulence factors associated with *Sporothrix* (García-Carnero and Martínez-Álvarez, 2022; Teixeira et al., 2014; Félix-Contreras et al., 2020). We downloaded the gene sequences of these virulence factors from the NCBI and utilized the JASPAR database^2^ to identify virulence factors containing AbaA binding sites (Andrianopoulos and Timberlake, 1994). The sequences of the *abaA* binding sites were then compared using DANMAN sequence alignment software, allowing us to screen for virulence factors that may be regulated by the *abaA* gene. These genes are GPI-anchored cell wall beta-1,3-endoglucanase EglC, scytalone dehydratase, CFEM domain protein, laccase precursor, molecular chaperone HTPG, DNA mismatch repair protein. This screening was subsequently validated through transcriptome analysis. To validate the reliability of the bioinformatics analysis, we subsequently conducted transcriptome analysis and qRT-PCR for verification.

### 3.6 Transcriptome sequencing data processing and assembly result statistics

RNA-Seq sequencing yielded between 41,934,980 and 47,331,900 raw reads from nine libraries, with raw bases ranging from 6.29 G to 7.02 G. The number of clean reads varied from 43,514,694 to 8,516,234. The GC content ranged from 54.26 to 56.27%. The Q30(%), which represents the percentage of bases with an accuracy exceeding 99.9%, the Q30 value was greater than 92% in this experiment, indicating that the sequencing quality was reliable (Supplementary Table S5).

**Figure 7:**
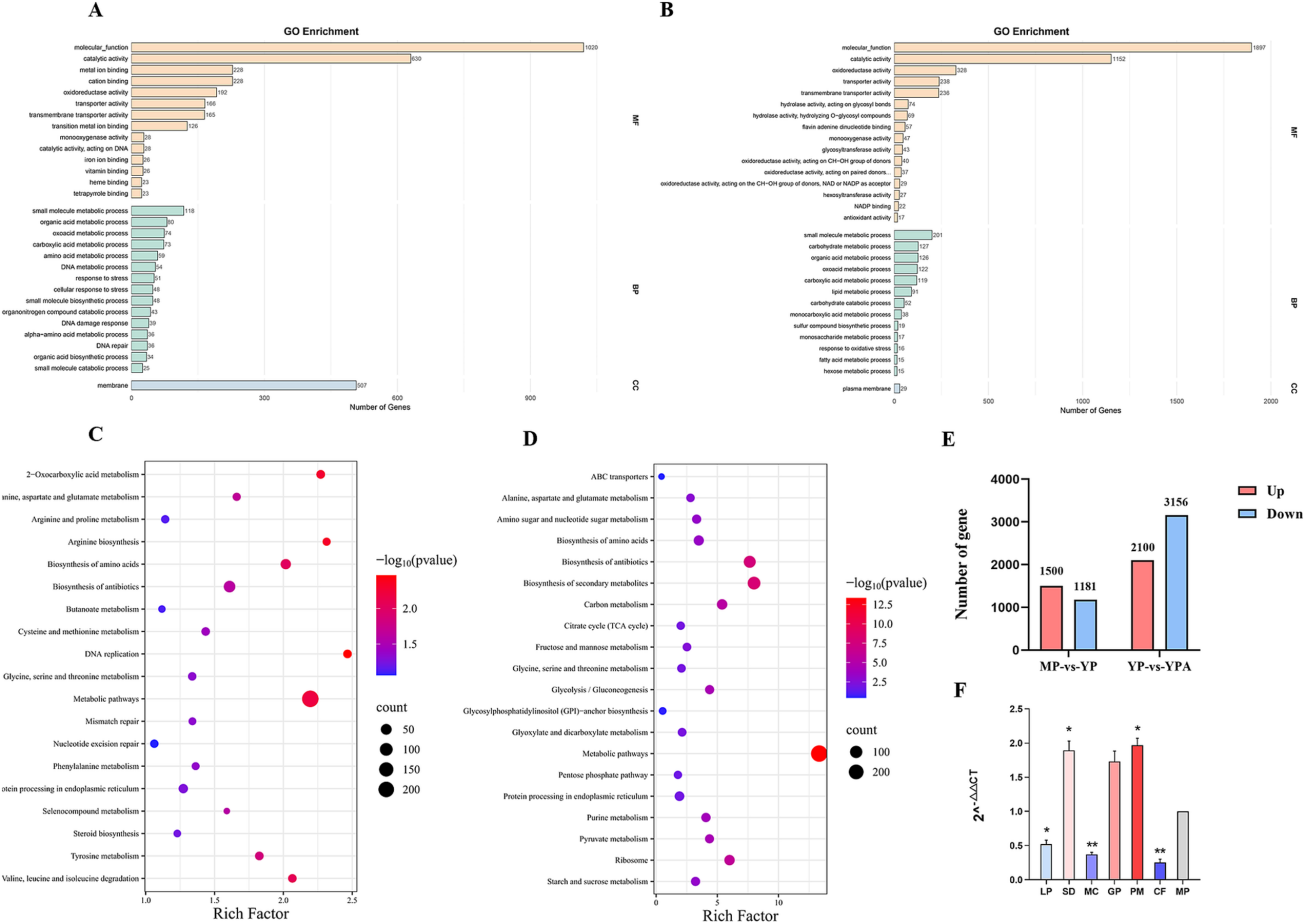
Transcriptome data analysis. **(A)** Gene Ontology Enrichment Analysis of MP and YP differentially expressed genes. **(B)** Gene Ontology Enrichment Analysis of YP and YPA differentially expressed genes. **(C)** Histogram of the Kyoto Encyclopedia of Genes and Genomes pathway classification of differentially-expressed genes of MP and YP. **(D)** Histogram of the Kyoto Encyclopedia of Genes and Genomes pathway classification of differentially-expressed genes of YP and YPA. **(E)** Histograms of differentially expressed genes. There were 2,681 genes differing between MP and YP, and 5,256 genes differing between YP and YPA. **(F)** qRT-PCR showed that *abaA* regulated virulence factor gene was expressed in yeast phase and mycelial phase with other genes. The expression of each gene was expressed as 2^-△△ CT value (mean ± SEM), and the expression of each gene in the mycelial phase was constant as 1, which served as a baseline comparison with other genes in the yeast phase. Gene: LP: laccase precursor; SD: scytalone dehydratase; MC: molecular chaperone HtpG; GP: GPI-anchored cell wall beta-1,3-endoglucanase EglC; PM: DNA mismatch repair protein. CF: CFEM domain protein. The significance was analyzed by independent sample *t* test (*, *p* < 0.05; **, *p* < 0.01). MP: mycelial phase baseline.

Gene expression was analyzed using DESeq, with the criteria for screening differentially expressed genes set at |log2foldchange| > 1 and significance *p* < 0.05. A total of 2,681 genes were found to be differentially expressed between the Mycelial Phase (MP) and Yeast Phase (YP), of which 1,500 genes were up-regulated and 1,181 genes were down-regulated. Additionally, 5,256 genes were differentially expressed between Yeast Phase + Azelastine (YPA) and Yeast Phase (YP), with 2,100 genes up-regulated and 3,156 genes down-regulated (Figure 7E).

We utilized the Gene Ontology (GO) and Kyoto Encyclopedia of Genes and Genomes (KEGG) databases to analyze significantly differentially expressed genes between the MP and YP, as well as between the YP and YPA. The GO functional enrichment analysis for the comparison of the MP versus YP (Figure 7A) revealed that 1,020 differentially expressed genes were enriched in molecular functions, with 630 genes enriched in catalytic activity and 507 genes enriched in membrane functions. The KEGG pathway enrichment analysis (Figure 7C) indicated that the differentially expressed genes in the treatment group were significantly enriched in metabolic and biosynthetic pathways, such as valine, leucine, and isoleucine degradation (KEGG: ko00280) and amino acid biosynthesis (KEGG: ko01230). The enrichment of these differential genes in the aforementioned functions and pathways suggests potential changes in cell membrane structure and the production of virulence factors during the dimorphic transformation of *Sporothrix*. Notably, serine metabolism can generate precursors for glutathione, enhancing the fungus’s ability to cope with reactive oxygen species (ROS) produced by the host’s immune response, thereby aiding its survival following macrophage phagocytosis (Cheng et al., 2019).

For the YP and YPA, GO functional enrichment analysis (Figure 7B) revealed that 1,897 differentially expressed genes were enriched in molecular functions, with 1,152 genes enriched in catalytic activity and 328 genes enriched in oxidative activity. Additionally, KEGG pathway enrichment analysis (Figure 7D) demonstrated that the differentially expressed genes in this treatment group were enriched in the biosynthesis of secondary metabolites (KEGG: ko01110) and metabolic pathways. The significant enrichment of differentially expressed genes in molecular functions, catalytic activity, and metabolic pathways indicates that the drug had a substantial impact on the original metabolic activities of the strain. Furthermore, the functional enrichment of differentially expressed genes in oxidative activity and the biosynthesis of secondary metabolites pathway suggests that the drug may have exerted specific effects on the synthesis of secondary metabolites and the oxidative stress system in the fungal. This further implies potential disturbances in the synthesis of virulence factors and the mechanisms of host immune evasion in the fungal. Transcriptome results indicated that several virulence factors regulated by *abaA*, including GPI-anchored cell wall beta-1,3-endoglucanase (EGLC), dehydratase, and CFEM domain protein, were significantly down-regulated. Conversely, genes associated with fungal growth and repair, such as laccase precursor, molecular chaperone HTPG, and DNA mismatch repair protein, exhibited significant up-regulation. Further validation and functional analyses of these candidate genes will be performed.

### 3.7 The results of qRT-PCR were consistent with those of transcriptome

**Figure 8:**
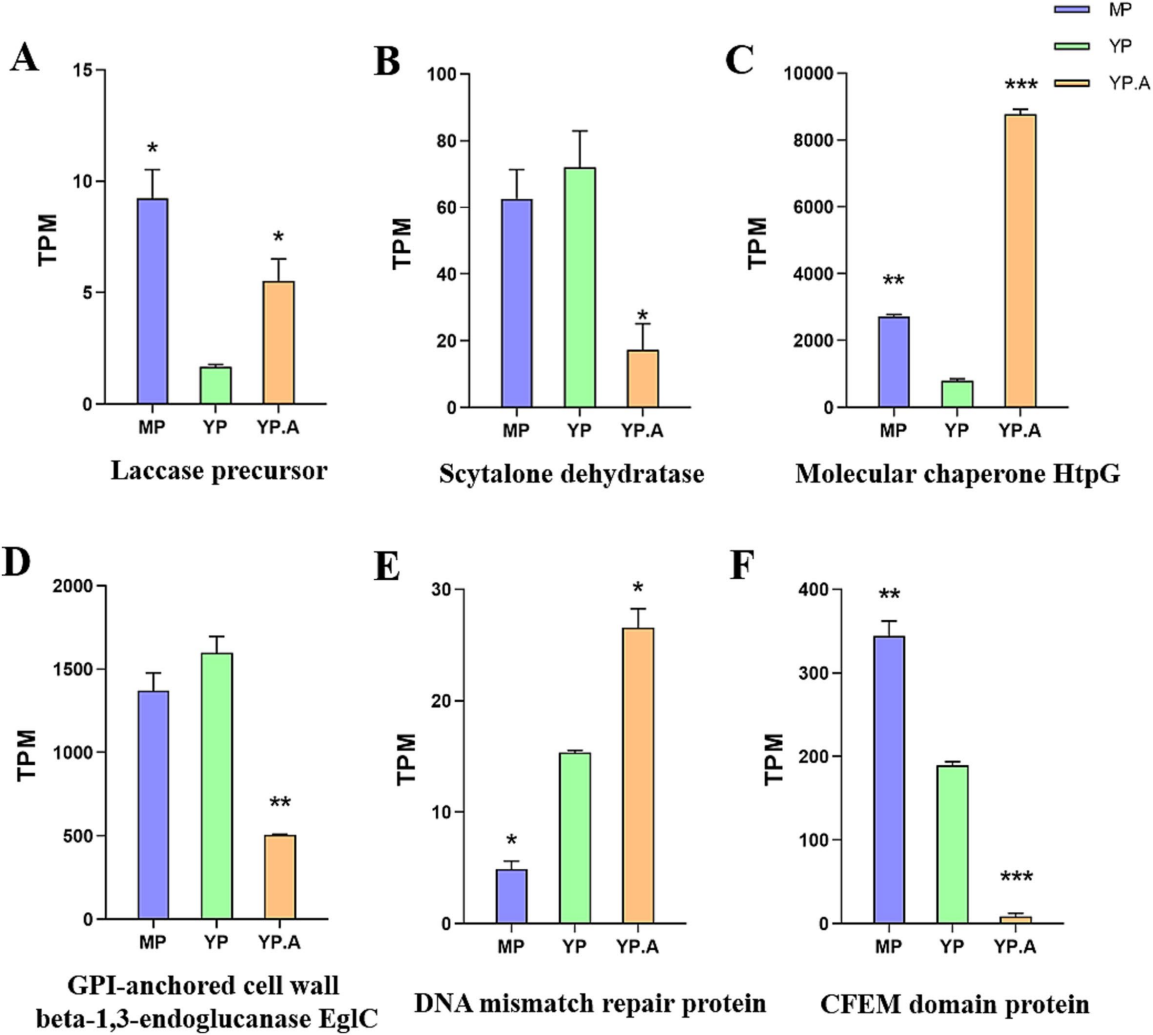
**(A-F)** The transcriptome shows the ratio of *abaA*-regulated virulence factors to other genes in mycelial phase, yeast phase, and yeast phase + Azelastine, with results presented as TPM values (mean ± SEM), significance was tested by independent-sample *T*-test with yeast phase as baseline (*, *p* < 0.05; **, *p* < 0.01; ***, *p* < 0.001).

To verify the reliability of the transcriptome results, we performed qRT-PCR validation. (Figure 7F). The primers used are shown in Supplementary Table S4. The results of qRT-PCR were consistent with those of transcriptome analysis (Figure 8). Interestingly, among the screened genes, genes related to the cell wall structure of *Sporothrix* (GPI-anchored cell wall beta-1,3-endoglucanase EglC, CFEM domain protein) were significantly down-regulated; and genes related to melanin (scytalone dehydratase) were significantly down-regulated. Other genes such as DNA repair-related genes (molecular chaperone HtpG, DNA mismatch repair protein) and fungal adaptability-related genes (laccase precursor) were significantly up-regulated. This suggests that the *abaA* gene plays a crucial role in regulating the anchoring of the *Sporothrix* cell wall to the host matrix and in the regulation of melanin. Furthermore, inhibition of the *abaA* gene results in a significant expression of repair genes, highlighting its importance to *Sporothrix*. We analyzed the transcriptome data of the virulence factors regulated by the *abaA* gene and observed that the expression levels of these virulence factors were inversely correlated with those of yeast following the addition of Azelastine (Figure 8). This finding further confirms that the expression level of *abaA* is down-regulated upon the introduction of small molecule drugs, indicating that the *abaA* gene serves a regulatory function in these virulence factors.

## 4 Discussion

Currently, the issue of drug resistance in invasive fungal diseases is becoming increasingly serious, with emerging resistance also observed in sporotrichosis. Therefore, there is an urgent need to search for new small-molecule drugs to treat sporotrichosis and to explore innovative methods for drug discovery. If we want to find new small molecule drugs, we have to find new targets (Ortiz-Ramírez et al., 2022; Cortés Juan Carlos et al., 2019). *S. globosa* is a dimorphic fungus that exists in the mycelial form in the environment at 25°C, where it is non-pathogenic. Upon entering the body at 37°C, *S. globosa* transforms into its yeast phase, at which point it becomes pathogenic. The AbaA gene, which is involved in the dimorphic switching process, can influence the virulence of *S. globosa*, making it a key target for selection. The *abaA* gene, which plays a crucial role in the dimorphic switch process, can influence the virulence of *S. globosa*, making it a focal point for target selection. The *abaA* gene is essential for dimorphic switch; in *Talaromyces marneffei*, deletion of the *abaA* gene impacts dimorphic switch (Borneman et al., 2000), while in *S. schenckii*, deletion of the *abaA* gene affects virulence. Consequently, we chose *abaA* gene as a novel drug target.

We employed molecular docking techniques to screen for small molecules that could inhibit *S. globosa* and *S. schenckii*. The literature review revealed that almost all invasive mycoses can lead to central nervous system diseases when they are severe (Krysan, 2016). Consequently, it is essential for fungicides to penetrate the blood–brain barrier and achieve elevated serum and tissue concentrations (Bing et al., 2012; Karbwang and Harinasuta, 1992). Through the analysis and screening of molecular docking results, we eliminated the side effects associated with drugs used for treating cancer and psychiatric diseases. Subsequently, we combined considerations of price, efficacy, and *in vitro* antifungal tests to identify two small-molecule drugs.

Two small-molecule drugs demonstrated a tendency to inhibit the growth of *Sporothrix* in antifungal experiments. The proposed mechanism involves the regulation of conidiophore development by AbaA. In *ΔabaA* mutants, the formation of the peduncle is associated with inhibited growth of *Sporothrix*, leading to the cessation of the developmental program. Furthermore, the molecular structures of the drugs Azelastine and Mefloquine interact with AbaA, resulting in the inhibition of both mycelial and spore development of *Sporothrix*, as evidenced by a decrease in OD_625_.Azelastine is a histamine 1 receptor blocker, its role is not an antagonist, but a reverse agonist, reduce the H1 receptor component activity (Watts et al., 2019). Azelastine has few side effects, is low-cost, can be taken for long periods of time and is safe for children over 6 years of age (Konrat et al., 2022). The drug has also been shown to have in vitro antiviral activity against the coronavirus (Lythgoe and Middleton, 2020). In addition, Azelastine also has anti-inflammatory effects, mainly by stabilizing mast cells and inhibiting the production of leukotrienes and proinflammatory cytokine (Watts et al., 2019). It can also down-regulate the expression of intercellular adhesion molecule-1 and reduce the migration of inflammatory cells. However, the effects of Azelastine on fungi have been poorly studied. Azelastine, a cationic amphiphilic drug belonging to a pharmacologically diverse class of compounds with distinct target molecules (Tummino et al., 2021), has been shown to induce phospholipidosis at the submicron scale. This property suggests that its antifungal activity against *Sporothrix* may result from a combination of direct fungal growth inhibition and host-mediated mechanisms. Furthermore, Azelastine remains effective when administered as a nasal spray for the treatment of rhinitis, indicating its significant potential as a nasal spray for the treatment of sporotrichosis (Konrat et al., 2022).

The results of pharmacological studies of Mefloquine show that Mefloquine has many useful features in the treatment of fungal infections. First, when taken orally, it can be well absorbed and establishes high serum and tissue levels, second, it can penetrate the blood–brain barrier to high nerve concentrations relative to plasma, Mefloquine has a long half-life, so it can be used as a prophylactic regimen (Montoya et al., 2020). In addition, some studies have shown that Mefloquine has certain antifungal activity against *Candida*, *Cryptococcus* and *Aspergillus* (Montoya et al., 2020). Mefloquine has been shown to inhibit the formation of egg granulomas in *Schistosoma japonicum*, suggesting that it may play a role in modulating the host immune system *in vivo* (Huang et al., 2011). Furthermore Mefloquine may inhibits *Sporothrix* through two mechanisms, in addition to binding to key target proteins of *Sporothrix*, it may also influence the host immune response. The underlying mechanisms of this interaction will be explored in further studies. The selection of dosing concentration was informed by a review of pertinent literature, indicating that both Azelastine and Mefloquine are capable of penetrating the blood–brain barrier and possess potential therapeutic effects against fungal infections. With regard to Azelastine, The pharmacokinetic results of the drug demonstrated Azelastine 16 mg did not increase side effects, and the maximum dose for adults was 40 mg, through the above pharmacokinetics analysis, combined with mouse and human drug delivery conversion formula, we selected two dosing concentrations of 3 mg/kg and 6 mg/kg, to conduct animal experiments (McTavish and Sorkin, 1989). About Mefloquine, adults were given 18–20 mg/kg, or (750–1,250 mg) a day, the clinical pharmacokinetics of Mefloquine showed that the peak time of Mefloquine administration was 6–24 h in healthy subjects, the maximum blood concentration of the drug was 1,000 μg/mL after taking 1,000 mg. Based on the above pharmacokinetics analysis, we selected a dosing concentration of 3.8 mg/kg; we also found that many studies treated neuropathic pain by intraperitoneal administration of Mefloquine at a dose of 20 mg/kg, so we chose a larger concentration of 20 mg/kg to study the effect of Mefloquine on sporotrichosis. As for the choice of the positive itraconazole concentration, the final choice was 60 mg/kg according to the conversion of doses between human and mice (Bonifaz and Vázquez-González, 2010; Saraiva et al., 2012; Moreira et al., 2015).

The *abaA* gene is crucial for the dimorphic switch of *Sporothrix* and influences the virulence of certain fungi. In this study, several genes regulated by *abaA*, including various virulence factors and functional genes, were identified through a combination of bioinformatics predictions and experimental approaches. GPI-anchored cell wall beta-1,3-endoglucanase EGLC functions as a GPI-anchored protein. Its biosynthesis is crucial for maintaining the integrity of fungal cell walls and is recognized as a significant virulence factor in various pathogenic fungi, including *Candida albicans*, where it plays an adhesive role in the process of host cell infection (Martinez-Lopez et al., 2004; Cormack et al., 1999). It is also an important target for many fungicide. The CFEM domain protein, characterized by its unique cell wall structure in fungi, is associated with glycosylphosphatidylinositol (GPI)-anchored proteins. This protein can bind to virulence factor effectors that contain the CFEM domain, thereby playing a critical role in the formation and enhancement of adhesion and virulence (Choi and Dean, 1997; Wang et al., 2022). As a classical virulence factor of pathogenic fungi, melanin plays a significant role in the immune evasion of these organisms, particularly in their resistance to oxidative attacks from immune cells, such as reactive oxygen species (ROS) (García-Carnero and Martínez-Álvarez, 2022; Wang et al., 1995). Scytalone dehydratase has been implicated in the formation of melanin virulence factors in fungi. After Wang et al. expressed the gene for scytalone dehydratase, which encodes the sickle dehydratase, in melanin-deficient, nonpathogenic *Colletotrichum lagenarium* (OSD1), it restored both melanin production and pathogenicity in this species (Wang et al., 2001). Furthermore, scytalone dehydratase has been utilized as a target for antifungal drug development aimed at inhibiting melanin synthesis (Eisenman et al., 2009). Laccase precursor is a precursor of laccase (Motoyama et al., 2022), a virulence-related cell wall enzyme associated with the blackening of *Cryptococcus neoformans* and the acquisition of resistance to polyene and echinocandins (Eisenman et al., 2009). The DNA mismatch repair protein- PMS2 is responsible for recognizing and repairing erroneous insertions, deletions, and base misincorporations that occur during DNA replication and recombination, as well as for addressing certain types of DNA damage (Iyer et al., 2006). Its absence contributes to the evolution of fungal resistance (Legrand et al., 2007; Dos Reis et al., 2019). Molecular Chaperone HTPG, a member of the Hsp90 family, is an important chaperone whose overexpression enhances the virulence of *Candida* in mice (Hodgetts et al., 1996). It is also considered central to the buffering effect known as pipelization (Burnie et al., 2006). HTPG has been described as suppressing phenotypic variation under normal conditions, yet it releases such variation when its function is impaired (Bergman and Siegal, 2003). Therefore, these genes may serve as new targets for future research.

## 5 Conclusion

In this study, we employ bioinformatics to identify new therapeutic targets for Sporotrichosis. To further predict these targets, selected small molecule drugs were tested *in vitro* and *in vivo*. The results concluded that Azelastine and Mefloquine are effective in treating Sporotrichosis. Furthermore, our study indicates that these small molecule drugs may possess a broad-spectrum antifungal effect, potentially providing new insights for the treatment of other invasive fungal diseases. At the same time, we investigated the virulence factors and growth repair-related genes regulated by *abaA* through transcriptomics and qRT-PCR. The results indicated that *abaA* is crucial for the toxicity, growth, and development of *Sporothrix*. The methodology employed in this research establishes a foundation for the investigation of drug resistance in other fungi, and the regulation of downstream related factors by *abaA* offers a robust basis for future studies.

## Data Availability

The datasets presented in this study can be found in online repositories. The names of the repository/repositories and accession number(s) can be found at: NCBI PRJNA1163991.

## Footnotes

^1^ https://robetta.bakerlab.org/

^2^ https://JASPAR.elixir.no/
