# Supplementary material for: Screening and transcriptomic analysis of anti-Sporothrix globosa targeting AbaA
Source: Front Microbiol. 2025 Apr 29;16:1546020. doi: 10.3389/fmicb.2025.1546020 (PMC12069444; doi:10.3389/fmicb.2025.1546020)
Supplement: Supplementary file 1 [file Data_Sheet_1.docx]

Supplementary Material

# Supplementary Figures


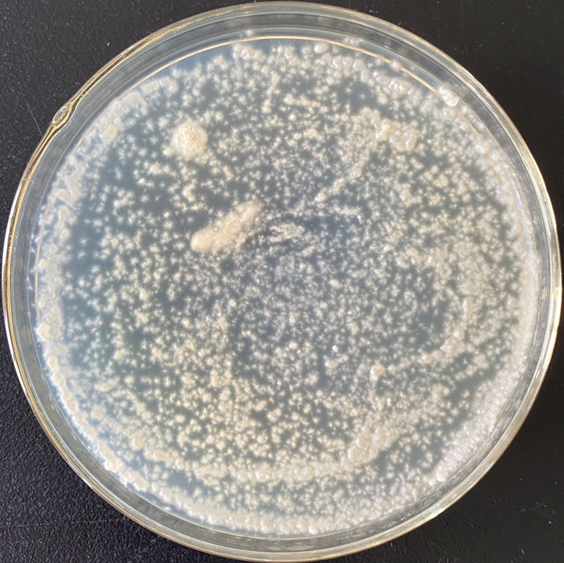


**Supplementary Figure 1.** Results of purulent culture in mice. The purulent fluid obtained from the skin lesion of mice was cultured and proved to be sporothrix, which proved that the model was successful.

# Supplementary Tables

Table S1. Protein retrieval results.

| Entry | Entry  Name | Protein  Names | Gene  Names | Organism | Length |
| --- | --- | --- | --- | --- | --- |
| U7PZ06 | U7PZ06_SPOS1 | TEA domain-containing protein | HMPREF1624_03084 | *Sporothrix schenckii* (strain ATCC 58251 / de Perez 2211183) | 1,120 AA |

Table S2. AbaA protein DNA binding domain.

| DNA domain | Location | Amino acid sequences |
| --- | --- | --- |
| TEA | 235-321 | DDKTEQIWPDVLEDAFLDALLLIPQMGRKKYAMRGQLHGRNMLISEYLWVAYCLSLPPGA  KPDRKMARGRKQVSSHIQVLKNFFIHH |

Table S3. Virtual Screening results

| Number | Common name | Formula | Binding Energy (Kcal/mol) | |
| --- | --- | --- | --- | --- |
| 1  2  3  4  5  6  7  8  9  10  11  12  13  14  15  16  17  18  19  20  21  22  23  24  25  26  27  28  29  30  31 | Lapatinib  Ergotamine  Olaparib  Trypan Blue  Dihydroergotamine  Vumon  Yaz  Isavuconazonium  Accolate  Lumacaftor  Azulfidine  Eltrombopag  Nilotinib  Mepron  Imatinib  Mestranol  Halcion  Pazopanib  Avodart  Enjuvia  Ibrutinib  Celsentri  Alprazolam  Naldemedine  Nilotinib  Trypan Blue  Rolapitant  Sqv  Mefloquine  Azelastine  Asenapine | C_29_H_26_ClFN_4_O_4_S  C_33_H_35_N_5_O_5_  C_24_H_23_FN_4_O_3_  C_34_H_28_N_6_O_14_S_4_  C_33_H_37_N_5_O_5_  C_32_H_32_O_13_S  C_24_H_30_O_3_  C_35_H_35_F_2_N_8_O_5_S^+^  C_31_H_33_N_3_O_6_S  C_24_H_18_F_2_N_2_O_5_  C_18_H_14_N_4_O_5_S  C_25_H_22_N_4_O_4_  C_28_H_22_F_3_N_7_O  C_22_H_19_ClO_3_  C_29_H_31_N_7_O  C_21_H_26_O_2_  C_17_H_12_Cl_2_N_4_  C_21_H_23_N_7_O_2_S  C_27_H_30_F_6_N_2_O_2_  C_18_H_22_O_5_S  C_25_H_24_N_6_O_2_  C_29_H_41_F_2_N_5_O  C_17_H_13_ClN_4_  C_32_H_34_N_4_O_6_  C_28_H_22_F_3_N_7_O  C_34_H_28_N_6_O_14_S_4_  C_25_H_26_F_6_N_2_O_2_  C_38_H_50_N_6_O_5_  C_17_H_16_F_6_N_2_O  C_22_H_24_ClN_3_O  C_17_H_16_ClNO | -8.8  -8.7  -8.5  -8.5  -8.5  -8.4  -8.3  -8.3  -8.3  -8.2  -8.2  -8.2  -8.2  -8.2  -8.2  -8.1  -8.1  -8.1  -8  -8  -8  -8  -8  -8  -8.9  -8.7  -8.3  -8.2  -8.2  -8.1  -8.1 |  |

Table S4. Primer sequences

| **Gene name** | **Name of primer** | **Primer sequences** |
| --- | --- | --- |
| laccase precursor | SPSK 01067-F | CCGATGGTGAGGTCAAGGA |
|  | SPSK 01067-R | TGGGCTGAGAAGTGCGAGT |
| scytalone dehydratase | SPSK 06530-F | CGGCGTCTGGAAGTTTGC |
|  | SPSK 06530-R | GGTGTCTTGGGCACGGAAT |
| molecular chaperone HtpG | SPSK 08698-F | CCAATGCCTCTGATGCTC |
|  | SPSK 08698-R | CAACCAGGTAAGCCGAGTA |
| GPI-anchored cell wall beta-1,3-endoglucanase EglC | SPSK 01694-F | CCAGGGCTTCAACTACGGC |
|  | SPSK 01694-R | CCAGCATCACCACCAGACG |
| DNA mismatch repair protein PMS2 | SPSK 04415-F | CGAGGTCTACCGCTCCTACAA |
|  | SPSK 04415-R | GCACAGTAATGTCCTGCTTCTCA |

Table S5. Sequencing data statistics.

| Sample | Raw reads | Raw bases(G) | Clean reads | Q30(%) | GC content(%) |
| --- | --- | --- | --- | --- | --- |
| MP.1 | 44351228 | 6.65 | 41730130 | 94.18 | 55.93 |
| MP.2 | 45853744 | 6.88 | 43250686 | 93.61 | 56.15 |
| MP.3 | 46784164 | 7.02 | 43452070 | 93.32 | 55.69 |
| YP.1 | 46492690 | 6.97 | 43514694 | 93.54 | 56.21 |
| YP.2 | 41934980 | 6.29 | 39044244 | 92.99 | 56.27 |
| YP.3 | 46790950 | 7.02 | 43370230 | 93.3 | 56.14 |
| YP.A.1 | 45337102 | 6.8 | 8516234 | 94.05 | 54.26 |
| YP.A.2 | 47331900 | 7.1 | 9186834 | 93.86 | 54.45 |
| YP.A.3 | 46728680 | 7.01 | 8794122 | 93.71 | 54.63 |
